# Provider perspectives on the management of hidradenitis suppurativa in pregnancy – A survey study

**DOI:** 10.1016/j.ijwd.2020.12.002

**Published:** 2020-12-11

**Authors:** Erin K. Collier, Kyla N. Price, Tristan Grogan, Jennifer M. Fernandez, Justine R. Seivright, Raed Alhusayen, Afsaneh Alavi, Iltefat H. Hamzavi, Michelle A. Lowes, Martina J. Porter, Vivian Y. Shi, Jennifer L. Hsiao

**Affiliations:** a David Geffen School of Medicine, University of California, Los Angeles, CA, United States; b College of Medicine, University of Illinois, Chicago, IL, United States; c Department of Medicine Statistics Core, David Geffen School of Medicine, University of California, Los Angeles, CA, United States; d University of Arizona, College of Medicine, Tucson, AZ, United States; e Division of Dermatology and Sunnybrook Research Institute, University of Toronto, Ontario, Canada; f Division of Dermatology, Women College Hospital, University of Toronto, Toronto, Ontario, Canada; g Department of Dermatology, Henry Ford Hospital, Detroit, MI, United States; h The Rockefeller University, New York, NY, United States; i Department of Dermatology, Beth Israel Deaconess Medical Center and Harvard Medical School, Boston, MA, United States; j Department of Dermatology, University of Arkansas for Medical Sciences, Little Rock, AR, United States; k Division of Dermatology, Department of Medicine, David Geffen School of Medicine, University of California, Los Angeles, CA, United States

**Keywords:** Biologic medications, Hidradenitis suppurativa, Medical management, Pregnancy, Providers perspectives

Dear Editors,

Current treatment options for hidradenitis suppurativa (HS) include medical therapies, such as topical therapeutics, systemic antibiotics, oral retinoids, hormonal treatments, biologics, and immunosuppressants, as well as various procedural interventions (Alikhan et al., 2019). Treatment regimens need to be modified for pregnant patients due to safety concerns. However, there is a lack of expert consensus on evidence-based guidelines for management of HS in pregnancy (Adelekun et al., 2020). Herein, we investigate the perspectives and practice patterns of HS specialists regarding HS and pregnancy.

An anonymous questionnaire was distributed to online listservs of the United States and Canadian HS foundations. The study was exempt from University of Arizona institutional review board review. Statistical analyses were performed using IBM SPSS, version 25 (Armonk, NY). Spearman correlations (r_s_) were used to assess associations between variables; *p* < .05 was considered statistically significant.

**Fig. 1:**
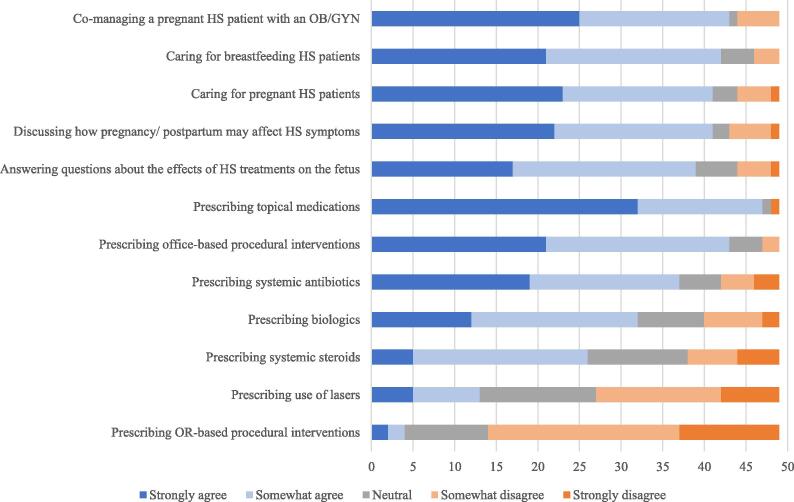
Provider perspectives on comfort level in managing pregnant patients with hidradenitis suppurativa (N = 49).

The demographics of the 49 physician respondents are summarized in Table 1. Nearly three-quarters of respondents (73%) were HS specialty clinic directors. The majority of respondents felt comfortable managing and counseling pregnant patients with HS (Fig. 1). Most respondents were comfortable prescribing topical medications (n = 47; 96%), systemic antibiotics (n = 37; 76%), biologics (n = 32; 65%), and systemic steroids (n = 26; 53%) and performing office-based procedures (n = 43; 88%) for pregnant patients with HS. Male respondents were more comfortable prescribing oral antibiotics (r_s_ = .378; *p* = .007) compared with their female counterparts. Providers with higher volumes of patients with HS were more comfortable with pregnant patients with HS receiving operating room-based procedures, such as those requiring general anesthesia or large wide local excisions that cannot be done in an office setting (r_s_ = .378; *p* = .007), or laser treatments (r_s_ = .429; *p* = .002) for HS compared with those with lower volumes. Directors of HS specialty clinics were also more comfortable with pregnant patients with HS receiving laser treatment (r_s_ = .366; *p* = .01) compared with non–HS specialty clinic directors.

**Table 1:** Survey respondent demographic information (n = 49).

Respondent characteristic	n (%)
Age (y)	
Mean ± standard deviation (range)	45.5 ± 12.5 (30–75)
Sex	
Male	27 (55)
Female	22 (45)
Country of practice	
United States	26 (53)
Canada	11 (23)
France	2 (4)
Spain	2 (4)
Brazil	2 (4)
Other*	6 (12)
Level of training	
Attending	44 (90)
Resident	3 (6)
Not specified	2 (4)
Years since completion of residency† (n = 44)	
Mean ± standard deviation (range)	12.7 ± 11.6 (1–46)
Average number of patients seen per month	
1–24	22 (45)
25–49	13 (27)
50–74	8 (16)
75–99	2 (4)
100+	4 (8)
Primary practice location	
Metropolitan	44 (90)
Rural	5 (10)
Primary practice setting	
Academic	37 (76)
Nonacademic	12 (24)
HS specialty clinic director	
Yes	36 (73)
No	13 (27)
Has prescribed or continued a biologic agent in a pregnant patient with HS	
Yes	29 (59)
No	20 (41)
Biologics prescribed or continued in pregnant patients with HS (n = 29)	
Adalimumab	26 (90)
Infliximab	12 (41)
Certolizumab	10 (34)
Secukinumab	1 (3)
Ustekinumab	1 (3)
General approach to managing a woman of childbearing age who is on a biologic for HS‡	
Keep patient on biologic throughout pregnancy	21 (43)
Discontinue biologic in third trimester	10 (20)
Discontinue biologic when patient is actively trying to get pregnant	8 (16)
Discontinue biologic upon finding out patient is pregnant	6 (12)
Discontinue biologic in second trimester	4 (8)

HS, hidradenitis suppurativa.

* Other countries include Belgium, Chile, Germany, Israel, Portugal, Saudi Arabia (each n = 1).

† Attendings only.

‡ If a patient has no preference or is seeking your recommendation, what is your general approach when managing a woman of childbearing age who is on a biologic for HS?

Additionally, 59% of participants reported that they have prescribed, or continued the use of, biologics for pregnant patients with HS. Those with a higher volume of patients with HS were more likely to have managed patients with HS with biologics during pregnancy (r_s_ = .321; *p* = .024) compared with those at clinics with a lower volume. Almost all biologics that respondents reported having prescribed to patients with HS during pregnancy were tumor necrosis factor-alpha inhibitors, including adalimumab, infliximab, and certolizumab. The timing of biologic use during pregnancy was mixed, with most respondents either keeping the patient on the biologic throughout pregnancy (43%) or discontinuing the biologic in the third trimester (20%; Table 1). No significant differences were observed based on sex, years of experience, or practice setting (academic vs. nonacademic).

HS specialists generally feel comfortable managing pregnant patients with HS; however, practice patterns for biologic use during pregnancy varied. Tumor necrosis factor-alpha inhibitors have more robust safety data in rheumatology and gastroenterology literature (Puchner et al., 2019). Interestingly, certolizumab use was reported in our study, even though its efficacy for HS is unclear (Porter et al., 2018). This may be because certolizumab’s molecular structure limits placental transfer (Mariette et al., 2018).

Response was mixed regarding comfort level with laser therapy for HS. Laser therapy has the advantage of avoiding systemic side effects and may be less risky than other therapeutic options. Identifying a provider with a higher volume of patients with HS or who directs an HS specialty clinic may be helpful in facilitating this procedure.

Study limitations include the small sample size and lack of inclusion of dermatologists who do not specialize in HS. Given the anonymous nature of the survey and the continuously changing numbers of participants in the provider listservs, the response rate is unknown. Our study underscores the need for evidence-based guidelines for management of HS in pregnancy. More data are needed on the safety and efficacy of medical and procedural interventions to treat pregnant women with HS.
